# The effects of ketogenic diet and calorie-restricted diet on metabolic dysfunction-associated steatotic liver disease: a retrospective study

**DOI:** 10.3389/fnut.2026.1790674

**Published:** 2026-05-05

**Authors:** Wei-jie Sun, Lin-li Xing, Su-fen Zhao, Hui-min Ying

**Affiliations:** 1 Office of Nutrition and Diet, Affiliated Hangzhou Xixi Hospital, Zhejiang Chinese Medical University, Hangzhou, China; 2 Department of Endocrinology, Affiliated Hangzhou Xixi Hospital, Zhejiang Chinese Medical University, Hangzhou, China

**Keywords:** body composition, hepatic steatosis, insulin sensitivity, low-carbohydrate diet, metabolic liver disease, nutritional therapy

## Abstract

**Objective:**

Metabolic dysfunction-associated steatotic liver disease (MASLD) is a prevalent condition with limited established dietary therapies. Lifestyle modifications, particularly dietary adjustments, represent the primary therapeutic approach for MASLD. This study investigated the effects of ketogenic diet (KD) and calorie-restricted diet (CRD) on hepatic fat accumulation and metabolic parameters in MASLD patients.

**Methods:**

This retrospective cohort study analyzed 102 MASLD patients completing 12-week nutritional management (KD or CRD). FibroTouch, anthropometric, body composition, and laboratory parameters were assessed at baseline and week 12.

**Results:**

After 12 weeks, the KD group achieved a significantly greater reduction in CAP (ΔCAP) compared to the CRD group (median ΔCAP: 62.0 dB/m (IQR: 48.0–78.5) vs. 36.0 dB/m (IQR: 26.0–52.0); *p* < 0.001, Cohen’s d = 0.93). A clinically meaningful reduction (≥40 dB/m) was achieved in 43 patients (84.3%) in the KD group, compared with 23 patients (45.1%) in the CRD group (*p* < 0.001). The proportion of patients with moderate-to-severe steatosis decreased more significantly in the KD group (68.6% vs. 39.2%, *p* < 0.05). No significant between-group difference was observed in liver stiffness measurement (LSM), although both groups showed significant within-group improvements. The KD group demonstrated significantly greater improvements in BMI, fat mass, body fat percentage, visceral fat grade, and waist-to-height ratio (all *p* < 0.05, Cohen’s d range: 0.44–1.07). However, the KD group also exhibited a significantly greater reduction in muscle mass compared to the CRD group (median *Δ*: 3.0 kg vs. 1.8 kg; *p* < 0.001, Cohen’s d = 0.59). Changes in body weight and waist-to-hip ratio were similar between groups. Among blood biomarkers, only fasting insulin showed a significantly greater reduction in the KD group (median *Δ*: 6.4 μIU/mL (IQR: 3.4–13.1) vs. 4.7 μIU/mL (IQR: 1.4–9.2); *p* < 0.05, Cohen’s d = 0.37). No significant between-group differences were observed in liver enzymes, lipid profile, fasting glucose, HOMA-IR, or inflammatory markers (all *p* > 0.05).

**Conclusion:**

KD significantly reduced hepatic steatosis and fasting insulin versus CRD, with comparable weight loss but greater muscle loss warranting caution. These findings support KD as a promising MASLD intervention, requiring confirmation in larger trials.

## 1 Introduction

Metabolic dysfunction-associated steatotic liver disease (MASLD) affects 30% of adults worldwide and is the leading cause of liver disease globally (1). Moreover, its prevalence continues to rise (2). MASLD is associated with insulin resistance, obesity, gut microbiota dysbiosis, and genetic risk factors. The obesity epidemic and type 2 diabetes contributed greatly to the increasing prevalence of MASLD (3). Several medications are being developed for MASLD, but none have been approved yet. Current management relies heavily on lifestyle interventions such as eating moderately, exercising more often, and weight loss (1, 4). Caloric restriction to attain weight loss (with or without exercise) can improve biomarkers of MASLD such as liver enzyme levels, steatosis, steatohepatitis, and fibrosis (5). Weight management is also a key strategy for MASLD (6). Hepatic steatosis, the primary feature of MASLD, arises from peripheral lipolysis driven by insulin resistance and hepatic *de novo* lipogenesis, transforming surplus carbohydrates into triglycerides. The buildup of intracellular hepatic lipids induces lipotoxicity, fostering inflammation, fibrosis, and the advancement of the disease (7, 8).

There is no consensus on the best diet for MASLD (9). The merits of different macronutrient diets are still unclear and not supported by large scale studies. In the past years low carbohydrate ketogenic diets have been used for obesity, type 2 diabetes and MASLD treatment (10). Although long term data comparing different weight loss regimens for MASLD are limited, many studies show that low carbohydrate diets lower triglycerides more than low fat, high carbohydrate diets (11). APASL has also suggested considering ketogenic diets as a possible intervention with weight loss and improved hepatic steatosis (12, 13). These results are supported by early small randomized controlled trials (14, 15). A network meta-analysis of 30 trials comparing various dietary patterns for MASLD revealed that low-carbohydrate diets ranked highest in improving both the controlled attenuation parameter (CAP) and body weight (16). Furthermore, studies indicate that ketogenic diets (KD) may provide weight-independent benefits by rapidly reducing liver fat, even prior to significant weight loss (17). The rapid reduction of hepatic fat is closely associated with diet-induced hormonal and metabolic substrate changes. Recent clinical evidence demonstrates that a 2-day isocaloric low-carbohydrate diet (with carbohydrates replaced by fats) can rapidly decrease hepatic fat content by 16% in overweight/obese individuals, whereas an equivalent carbohydrate-restricted very low-calorie diet fails to achieve this effect. This phenomenon suggests that macronutrient replacement to maintain energy balance inherently confers acute metabolic benefits. The core mechanism lies in the fact that carbohydrate restriction directly reduces circulating insulin levels and decreases glucose substrates utilized for hepatic fat synthesis. These two effects collectively effectively downregulate insulin-mediated *de novo* hepatic lipogenesis, resulting in a net reduction of hepatic fat storage within a short period (18). For patients with MASLD ketogenic diet (KD) may offer a mechanistically distinct approach to reducing hepatic steatosis beyond its effects on weight loss (4, 8, 19), though further investigation is needed to optimize its clinical application.

Both KD and CRD target hepatic fat reduction through energy restriction but differ in metabolic mechanisms and clinical implications. CRD relies on caloric deficit-driven weight loss to enhance insulin sensitivity and reduce hepatic fat gradually. It is established, accessible, safe, but may require longer intervention periods for significant outcomes (20). Conversely, KD induces rapid reduction in insulin levels and hepatic fat independent of caloric restriction through carbohydrate restriction. This suggests that KD may lead to quicker liver fat reduction before substantial weight loss (21). However, concerns exist about KD’s long-term sustainability, potential muscle loss, and impact on lipid profiles. Notably, our study highlights significant muscle loss with KD, emphasizing the importance of strategies to preserve muscle mass during its implementation (19).

Therefore, we compare directly KD versus calorie-restricted diet (CRD) effects on liver fat, insulin resistance, body composition and cardiometabolic parameters in MASLD patients. The results will help us design diets suitable for MASLD patients who want to lose weight and improve metabolic function.

## 2 Materials and methods

### 2.1 Study design and participants

This study is a single-center retrospective cohort study. Using the electronic medical record system and predefined inclusion criteria, 154 patients with MASLD who participated in nutritional interventions at the Fatty Liver Treatment Center of Hangzhou Xixi Hospital between October 2019 and June 2024 were identified. After applying exclusion criteria, 52 patients were excluded, and 102 patients were ultimately included in the analysis. Among them, 23 patients had an intervention duration of less than 12 weeks, primarily due to loss to follow-up and selection of alternative treatment regimens. Concurrently, we compared the baseline characteristics of these excluded intervention patients with those of the included cohort and found no statistically significant differences (all *p* > 0.05). This retrospective study was reviewed and approved by the Clinical Research Ethics Committee of Xixi Hospital, Hangzhou (Approval No.: 2025-Research Ethics-90) in accordance with the principles of the Declaration of Helsinki. As a retrospective study utilizing anonymized data, it complies with the ethics committee’s requirements for exemption and waiver of informed consent. Patients were included if they met all of the following criteria: (1) Age ≥ 18 years. (2) A diagnosis of MASLD, defined as: (a) Presence of hepatic steatosis, confirmed by either: (i) Imaging: Ultrasound, Controlled Attenuation Parameter (CAP) ≥ 248 dB/m, or MRI-PDFF ≥ 5.0%. (ii) Histology: Liver biopsy showing ≥5% macrovesicular steatosis. (b) Presence of at least two cardiometabolic risk factors: (i) Overweight or obesity (Body Mass Index, BMI ≥ 23 kg/m^2^ for Asian population). (ii) Prediabetes or type 2 diabetes. (iii) Blood pressure ≥130/85 mmHg or on antihypertensive therapy. (iv) Triglycerides ≥1.70 mmol/L or on lipid-lowering therapy. (v) HDL-C < 1.0 mmol/L (men) or <1.3 mmol/L (women). (3) Completion of a structured, supervised 12-week dietary intervention program with either KD or CRD, as documented by a clinical dietitian(4). (4) Availability of key data at both baseline and 12-week follow-up, including: (a) Hepatic steatosis quantification (CAP or equivalent). (b) Anthropometric measures (weight, BMI, waist circumference). (c) Fasting blood samples for insulin and liver enzymes. Patients were excluded if any of the following conditions were present: (1) Other specific etiologies of chronic liver disease: (a) Significant alcohol consumption (> 30 g/day for men, > 20 g/day for women). (b) Viral hepatitis (positive for HBsAg or HCV RNA). (c) Autoimmune liver disease, Wilson’s disease, or alpha-1 antitrypsin deficiency. (2) Use of medications known to significantly affect liver fat, weight, or glucose metabolism for more than 2 weeks during the intervention period, including: Glucagon-like peptide-1 receptor agonists (GLP-1 RAs). Sodium-glucose cotransporter-2 inhibitors (SGLT2i). Pioglitazone. (3) Medical conditions or histories that could independently influence body composition or study outcomes,such as a history of bariatric surgery, active malignancy or undergoing chemotherapy, severe renal or cardiovascular disease, or untreated thyroid dysfunction. (4) Conditions incompatible with the dietary interventions or affecting adherence: Pregnancy or lactation. Known allergy or intolerance to major components of the prescribed diets. Diagnosis of severe eating disorders. (5) Data insufficiency: Missing data for primary outcome at either baseline or 12-week follow-up.

### 2.2 Dietary intervention

Based on the patient’s clinical status, the clinical dietitian offered MASLD patients requiring nutritional intervention a choice between a ketogenic diet (KD) and a calorie-restricted diet (CRD). Clinical dietitians calculated the daily energy requirement by multiplying the estimated resting metabolic rate (eRMR) by the physical activity level (PAL) coefficient, daily energy intake was set at 70–80% of total energy requirements, approximately 1,300–1,500 kcal/day, distributed across three main meals and snacks. The eRMR is derived from the Mifflin prediction formula: (Male: 10 × body weight (kg) + 6.25 × height (cm) − 5 × age (years) + 5; Female: 10 × body weight (kg) + 6.25 × height (cm) − 5 × age (years) − 161). KD group: a daily dietary regimen in which 75% of energy is derived from fat, 20% from protein, and ≤5% from carbohydrates (≤50 g/day) (22). CRD group: Participants were advised to follow a balanced diet comprising 50–55% carbohydrates, 15% protein, and 25–30% fat (23).

To ensure intervention adherence, we implemented a multi-level monitoring and support system. All participants received lifestyle guidance from professional clinical dietitians, including regular meal schedules and sleep patterns. During the 12-week intervention period, participants were required to report daily dietary intake via a mobile application, with the KD group additionally reporting urinary ketone levels. Furthermore, we scheduled outpatient follow-ups every 2–4 weeks, where dietitians conducted structured assessments using food models combined with 24-h dietary recall methods, and calculated adherence scores (0–10 points) based on a comprehensive evaluation of intake of recommended food groups and achievement of macronutrient targets. A score ≥7 points was considered indicative of good adherence, and all assessment results were documented in medical records. The detailed scoring rubric is provided in Supplementary Appendix 1.

### 2.3 Data collection

#### 2.3.1 Data sources

The following data were collected from the hospital electronic medical record system, the laboratory information system, the body composition analysis system, and the imaging retrieval system.

#### 2.3.2 Baseline data

Height, weight, sex, age, body mass index (BMI), waist circumference (WC), hip circumference (HC), waist-to-height ratio (WHtR), waist-to-hip ratio (WHR).

#### 2.3.3 Laboratory parameters included

Liver enzymes (ALT, AST, GGT); lipid profile (TC, TG, HDL-C, LDL-C); glycemic indices (FPG, FINS, HOMA-IR); and other markers [UA, BUN, 25(OH)D, high-sensitivity C-reactive protein (hs-CRP)].

#### 2.3.4 Body composition indicators

Body fat percentage, body fat mass, muscle mass, visceral fat grade were measured by bioelectrical impedance analysis (TANITA MC-780MA, Japan).

#### 2.3.5 Imaging parameters

We utilized the Fibrotouch-B instrument (FT/B-001-002, Wuxi Haskell Medical Technology Co., Ltd.) to measure the patients’ liver stiffness measurement (LSM) and CAP. The severity assessment criteria for hepatic steatosis are as follows: Normal (based on the FibroTouch system’s built-in reference ranges): CA*p* value <240 dB/m; Mild: 240 dB/m ≤ CAP value <265 dB/m; Moderate: 265 dB/m ≤ CAP value <295 dB/m; Severe: CAP value ≥295 dB/m. The severity grading criteria for hepatic fibrosis are as follows: F0 (no hepatic fibrosis): LSM value <7.3 kPa; F1: 7.3 kPa ≤ LSM value <9.7 kPa; F2: 9.7 kPa ≤ LSM value <12.4 kPa; F3: 12.4 kPa ≤ LSM value <17.5 kPa; F4: LSM value ≥17.5 kPa.

Primary outcome: Degree of improvement in hepatic fat attenuation parameter after 12 weeks of intervention.

Secondary outcomes: changes in LSM; changes in body fat percentage, fat mass, muscle mass, and visceral fat grade; changes in glycemic metabolism indicators; and changes in liver enzymes and lipid profile.

### 2.4 Statistical analysis

Data analysis was performed using SPSS 26.0 software. The normality of continuous variables and their change scores (post-treatment minus pre-treatment) was assessed using the Shapiro–Wilk test. Normally distributed data were expressed as mean ± standard deviation (SD), while non-normally distributed data were presented as median and interquartile range (IQR). For baseline characteristics and intergroup comparisons, independent t-tests were used for normally distributed continuous variables, and Mann–Whitney U tests for non-normally distributed variables. Categorical variables were compared using chi-square tests or Fisher’s exact test. Within-group changes from baseline to week 12 were analyzed with paired t-tests for normally distributed data and Wilcoxon signed-rank tests for non-normally distributed data. Between-group change scores (*Δ*) were compared using independent t-tests or Mann–Whitney U tests as appropriate. For key secondary outcomes (muscle mass, CAP, and fasting insulin), covariance analysis (ANCOVA) was performed to adjust for baseline values. Week 12 values were designated as dependent variables, dietary groups as fixed factors, and corresponding baseline values as covariates. Adjusted mean differences were reported. The effect sizes of between-group comparisons for continuous variables were calculated using Cohen’s d, with Cohen’s d values of 0.2, 0.5, and 0.8 corresponding to small, moderate, and large effects, respectively. Due to the non-normal distribution of variables, Spearman’s rank correlation coefficient was used to analyze the relationship between waist-to-height ratio (ΔWHtR) changes and hepatic steatosis (ΔCAP) changes. Correlation analysis was performed in the overall cohort and separately for each dietary group. To investigate potential effect modifiers, subgroup analyses were conducted according to sex and baseline BMI. Interaction terms (group × subgroup) were included in a general linear model to test for statistical interactions. All tests were two-sided, with statistical significance set at *p* < 0.05.

## 3 Results

### 3.1 Baseline clinical parameters

**Figure 1:**
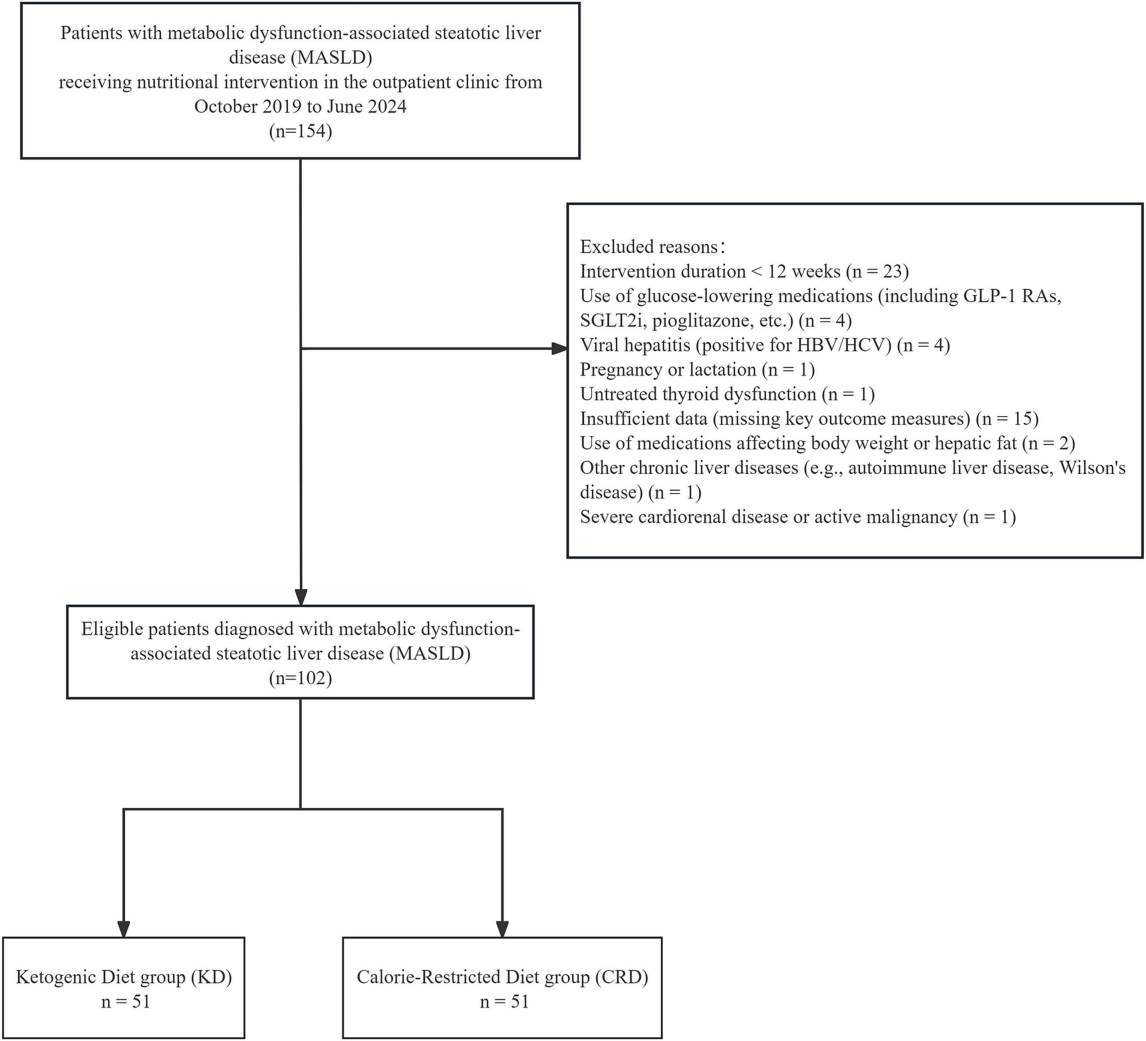
Patient flow diagram.

Among 154 initially screened patients, 52 were excluded for not meeting inclusion criteria, yielding a final study cohort of 102 patients (Figure 1). The mean age was 32.7 ± 8.9 years, body weight 86.4 ± 14.9 kg, BMI 31.4 ± 4.2 kg/m^2, WC 99.8 ± 10.5 cm, HC 107.7 ± 8.7 cm, WHR 0.9 ± 0.1, and WHtR 0.6 ± 0.1. At baseline, there were no significant differences between the KD and CRD groups in demographic characteristics, anthropometric measures, body composition, liver function, lipid profile, or glycemic indices (all *p* > 0.05) (Table 1).

**Table 1:** Baseline characteristics of patients in the ketogenic diet and calorie-restricted diet groups.

Indicator	CRD (*n* = 51)	KD (*n* = 51)	*p* value
Age, year	33.9 ± 11.6	31.4 ± 4.9	0.553
Gender (female/male)	22/29	24/27	0.691
Anthropometric measurements			
Height, cm	167.1 ± 8.67	167.9 ± 8.1	0.63
Weight, kg	80.8(77.2–94.4)	83.4(75.3–92.5)	0.965
BMI, kg/m^2^	29.5(27.5–33.6)	31.0(29.3–33.8)	0.153
WC, cm	99.1 ± 12.3	100.6 ± 8.2	0.463
HC, cm	106.0(98.0–112.0)	108.0(104.0–113.0)	0.099
WHR	0.93 ± 0.06	0.93 ± 0.06	0.813
WHtR	0.59 ± 0.07	0.60 ± 0.05	0.481
Anthropometric indices and body composition			
Body fat, %	35.4 ± 8.8	35.6 ± 8.4	0.915
Fat mass, kg	30.4(21.1–36.9)	29.5(25.7–34.9)	0.836
Muscle mass, kg	48.6(41.5–59.9)	48.5(42.7–61.3)	0.464
Visceral fat grade	12.8 ± 4.7	13.4 ± 3.7	0.431
Lipid profile			
TC, mmol/L	4.7 ± 0.9	5.0 ± 0.8	0.06
TG, mmol/L	1.6(1.3–2.2)	1.7(1.1–2.1)	0.579
LDL, mmol/L	2.8 ± 0.6	2.9 ± 0.6	0.312
HDL, mmol/L	1.1 ± 0.2	1.1 ± 0.2	0.235
Hepatic parameters			
ALT, U/L	37.0(23.0–64.0)	31.0(18.0–73.0)	0.725
AST, U/L	26.0(21.0–41.0)	24.0(18.0–48.0)	0.32
GGT, U/L	32.0(23.0–48.0)	33.0(22.0–47.0)	0.989
Glycemic indices			
FBS, mmol/L	5.4(5.0–6.2)	5.5(5.1–5.8)	0.843
FINS, μU/ml	13.5(9.2–18.4)	16.1(11.1–21.0)	0.167
HOMA-IR	3.4(2.3–4.9)	4.0(2.6–5.2)	0.252
Hepatic assessment via transient elastography			
LSM, kPa	7.5(6.2–9.6)	7.2(5.7–9.5)	0.34
CAP, dB/m	305.0(282.0–334.0)	312.0(296.0–323.0)	0.218
Moderate/ Severe	47(92.2%)	51(100%)	0.126
Degree of F0-1 of hepatic fibrosis	39(76.5%)	41(80.4%)	0.63
Other			
UA, μmol/L	419.8 ± 98.7	407.0 ± 95.5	0.505
BUN, mmol/L	4.5 ± 1.0	4.2 ± 1.0	0.269
hs-CRP, mg/L	1.6(0.8–4.7)	1.5(0.7–3.9)	0.987

CRD, calorie-restricted balanced diet; KD, ketogenic diet; BMI, body mass index; WC, waist circumference; HC, hip circumference; WHR, waist to hip ratio; WHtR, waist-to-height ratio; TC, total cholesterol; TG, triglycerides; LDL-C, low--density cholesterol; HDL-C, high-density cholesterol; ALT, alanine aminotransferase; AST, aspartate aminotransferase; GGT, gamma-glutamyl transferase; FBS, fasting blood sugar; FINS, fasting insulin; HOMA-IR, insulin resistance index; LSM, liver stiffness measurements; CAP, controlled attenuation parameter; UA, uric acid; BUN, blood urea nitrogen; hs-CRP, high-sensitivity C-reactive protein.

### 3.2 Effects on liver fibrosis and steatosis scores

**Figure 2:**
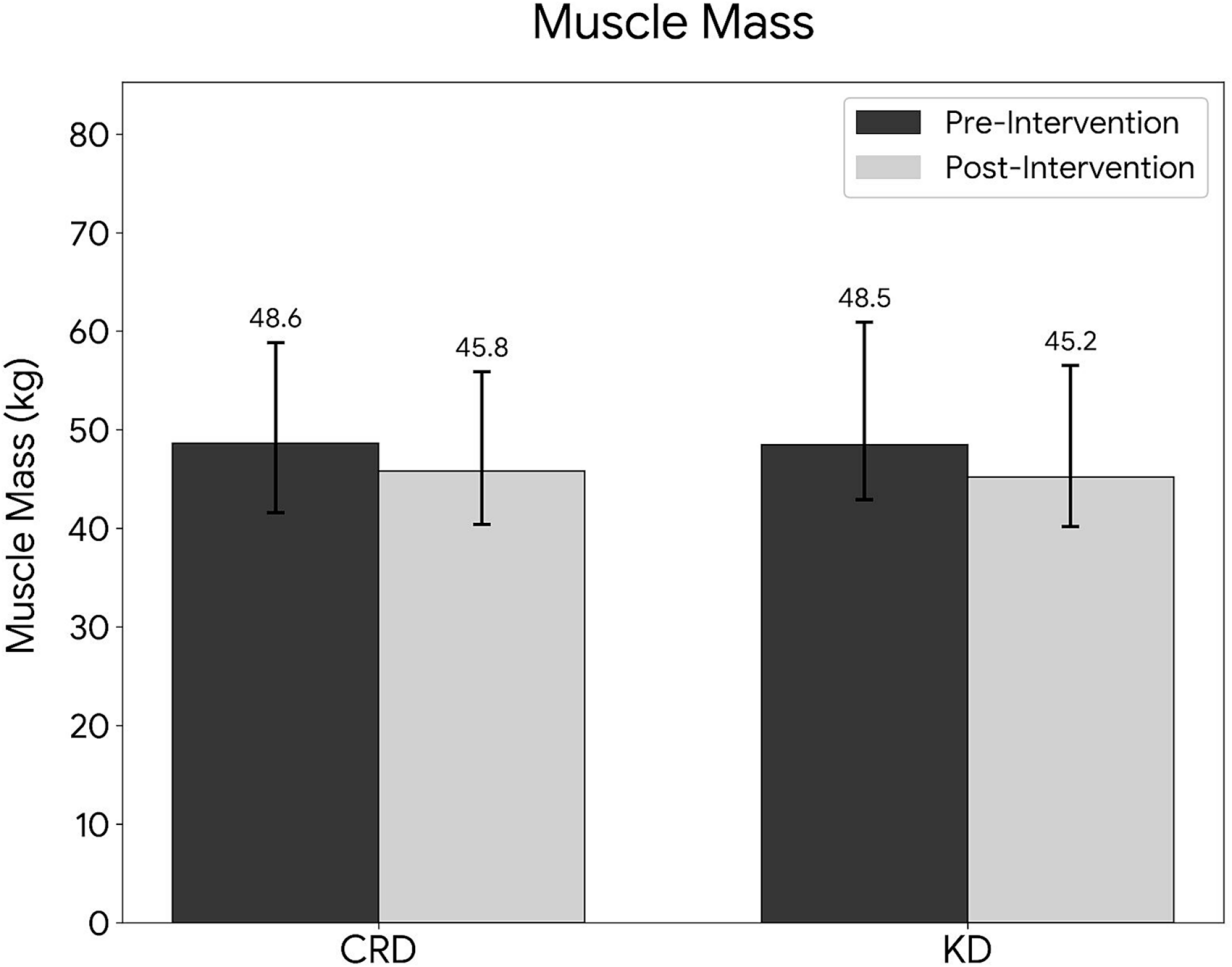
Comparison of CAP reduction between KD and CRD groups.

Compared with the CRD group, the KD group exhibited a significantly greater reduction in CAP values from baseline (median ΔCAP: 62.0 dB/m (IQR: 48.0–78.5) vs. 36.0 dB/m (IQR: 26.0–52.0); *p* < 0.001, Cohen’s d = 0.93, Figure 2). A clinically meaningful reduction (≥40 dB/m) was achieved in 43 patients (84.3%) in the KD group, compared with 23 patients (45.1%) in the CRD group (*p* < 0.001). The proportion of patients with moderate-to-severe steatosis decreased more significantly in the KD group than in the CRD group (68.6% vs. 39.2%; *p* < 0.05). No significant between-group differences were observed in LSM scores (*p* > 0.05) (Table 2), although both groups showed significant within-group LSM improvements (Table 3).

**Table 2:** Between-group comparisons of changes (Δ) from baseline to week 12 and effect sizes.

Indicator	Δ CRD (*n* = 51)	Δ KD (*n* = 51)	*P* value	Cohen’s *d*
Anthropometric measurements				
Weight, kg	9.9 (7.1–12.0)	10.0 (8.9–11.1)	0.656	0.05
BMI, kg/m^2^	3.6 ± 1.3	5.1 ± 1.6	<0.001**	1.00
WC, cm	8.3 ± 5.2	10.4 ± 4.2	0.030*	0.44
HC, cm	7.0 (3.5–9.0)	8.0 (6.0–12.0)	0.010**	0.59
WHR	0.02 ± 0.04	0.02 ± 0.04	0.924	0.02
WHtR	0.05 ± 0.03	0.06 ± 0.03	0.030*	0.44
Anthropometric indices and body composition				
Body fat, %	3.4 (1.8–4.5)	3.9 (2.9–5.9)	<0.001**	0.28
Fat mass, kg	4.5 (3.8–5.7)	6.9 (5.4–8.1)	0.020*	0.56
Muscle mass, kg	1.8 (0.9–3.1)	3.0 (2.2–3.9)	<0.001**	0.59
Visceral fat grade	3.0 (2.0–4.0)	4.0 (4.0–5.0)	<0.001**	1.07
Lipid profile				
TC, mmol/L	0.4 (−0.1–0.7)	0.3 (−0.1–1.0)	0.901	0.08
TG, mmol/L	0.5 ± 0.46	0.5 ± 0.8	0.802	0.05
LDL, mmol/L	0.3 ± 0.4	0.2 ± 0.5	0.228	0.24
HDL, mmol/L	0 (−0.1–0.1)	0 (−0.1–0.1)	0.745	0.12
Hepatic parameters				
ALT, U/L	12.0 (4.0–36.5)	13.0 (5.5–50.0)	0.63	0.19
AST, U/L	7.0 (3.0–14.0)	5.0 (1.0–20.0)	0.661	0.20
GGT, U/L	11.0 (5.0–22.0)	15.0 (6.0–30.0)	0.148	0.26
Glycemic indices				
FBS, mmol/L	0.3 (−0–0.7)	0.4 (0.1–0.7)	0.447	0.08
FINS, μU/ml	4.7 (1.4–9.2)	6.4 (3.4–13.1)	0.044*	0.37
HOMA-IR	1.2 (0.3–2.7)	1.6 (0.8–3.6)	0.071	0.34
Hepatic assessment via transient elastography				
LSM, kPa	1.2 (0.6–3.0)	0.6 (0.2–2.3)	0.146	0.25
CAP, dB/m	36.0 (26.0–52.0)	62.0 (48.0–78.5)	<0.001**	0.93
Moderate/severe	20 (39.2%)	35 (68.6%)	0.003**	
Degree of F0-1 of hepatic fibrosis	9 (17.6%)	6 (11.8%)	0.402	
Clinically meaningful CAP reduction (≥40 dB/m)	23 (45.1%)	43 (84.3%)	<0.001**	
Other				
UA, μmol/L	34.9 (6.0–92.2)	87.6 (30.3–122.1)	0.071	0.15
BUN, mmol/L	0 (−0.8–0.5)	0 (−0.6–1.0)	0.252	0.31
hs-CRP, mg/L	0.1 (−0.2–0.8)	0.2 (0–1.1)	0.482	0.17

Δ values represent change from baseline to week 12 (baseline – week 12); *p < 0.05 (between-group comparisons); **p < 0.01 (between-group comparisons); CRD, calorie-restricted balanced diet; KD, ketogenic diet; BMI, body mass index; WC, waist circumference; HC, hip circumference; WHR, waist to hip ratio; WHtR, waist-to-height ratio; TC, total cholesterol; TG, triglycerides; LDL-C, low--density cholesterol; HDL-C, high-density cholesterol; ALT, alanine aminotransferase; AST, aspartate aminotransferase; GGT, gamma-glutamyl transferase; FBS, fasting blood sugar; FINS, fasting insulin; HOMA-IR, insulin resistance index; LSM, liver stiffness measurements; CAP, controlled attenuation parameter; UA, uric acid; BUN, blood urea nitrogen; hs-CRP, high-sensitivity C-reactive protein.

**Table 3:** Summary of data from baseline to week 12 in the ketogenic diet group and calorie restriction diet group.

Indicator	KD (*n* = 51)			CRD (*n* = 51)		
	Baseline	Week 12	*P* value	Baseline	Week 12	*P* value
Anthropometric measurements						
Weight, kg	83.4 (75.3–92.5)	73.7 (65.0–81.4)	<0.001**	80.8 (77.2–94.4)	73.2 (66.2–86.5)	<0.001**
BMI, kg/m^2^	31.0 (29.3–33.8)	25.5 (24.4–28.2)	<0.001**	29.5 (27.5–33.6)	27.0 (23.6–30.3)	<0.001**
WC, cm	100.6 ± 8.2	90.3 ± 9.8	<0.001**	99.1 ± 12.3	90.8 ± 10.8	<0.001**
HC, cm	108.0 (104.0–113.0)	98.0 (95.0–101.0)	<0.001**	106.0 (98.0–112.0)	99.0 (94.0–104.0)	<0.001**
WHR	0.93 ± 0.06	0.91 ± 0.07	0.001**	0.93 ± 0.06	0.91 ± 0.07	0.001**
WHtR	0.60 ± 0.05	0.54 ± 0.06	<0.001**	0.59 ± 0.07	0.54 ± 0.06	<0.001**
Anthropometric indices and body composition						
Body fat, %	35.6 ± 8.43	31.7 ± 8.9	<0.001**	35.4 ± 8.8	32.4 ± 8.9	<0.001**
Fat mass, kg	29.5 (25.7–34.9)	22.0 (18.6–28.3)	<0.001**	30.4 (21.1–36.9)	26.2 (17.8–29.8)	<0.001**
Muscle mass, kg	48.5 (42.7–61.3)	45.2 (40.2–56.6)	<0.001**	48.6 (41.5–59.9)	45.8 (40.3–56.0)	<0.001**
Visceral fat grade	13.4 ± 3.7	8.9 ± 4.0	<0.001**	12.8 ± 4.7	9.9 ± 4.2	<0.001**
Lipid profile						
TC, mmol/L	5.0 ± 0.8	4.7 ± 0.9	0.006**	4.7 ± 0.9	4.3 ± 0.8	0.001**
TG, mmol/L	1.6 (1.3–2.2)	1.1 (0.9–1.5)	<0.001**	1.7 (1.1–2.1)	1.1 (0.9–1.6)	<0.001**
LDL, mmol/L	2.9 ± 0.6	2.7 ± 0.6	0.009**	2.8 ± 0.6	2.5 ± 0.6	<0.001**
HDL, mmol/L	1.1 ± 0.2	1.1 ± 0.2	0.761	1.1 ± 0.2	1.1 ± 0.2	0.124
Hepatic parameters						
ALT, U/L	31.0 (18.0–73.0)	16.0 (11.0–23.0)	<0.001**	37.0 (23.0–64.0)	19.0 (15.0–28.0)	<0.001**
AST, U/L	24.0 (18.0–48.0)	18.0 (15.0–21.0)	<0.001**	26.0 (21.0–41.0)	20.0 (17.0–25.0)	<0.001**
GGT, U/L	33.0 (22.0–47.0)	16.0 (14.0–21.0)	<0.001**	32.0 (23.0–48.0)	19.0 (14.0–29.0)	<0.001**
Glycemic indices						
FBS, mmol/L	5.5 (5.1–5.8)	5.0 (4.7–5.3)	0.001**	5.4 (5.0–6.2)	5.1 (4.8–5.4)	0.002**
FINS, μU/ml	16.1 (11.1–21.0)	7.0 (5.3–11.0)	<0.001**	13.5 (9.2–18.4)	7.6 (6.0–11.0)	<0.001**
HOMA-IR	4.0 (2.6–5.2)	1.5 (1.2–2.5)	<0.001**	3.4 (2.3–4.9)	1.8 (1.3–2.6)	<0.001**
Hepatic assessment via transient elastography						
LSM, kPa	7.2 (5.7–9.5)	5.9 (5.1–7.1)	0.005**	7.5 (6.2–9.6)	6.0 (5.4–7.0)	<0.001**
CAP, dB/m	312.0 (296.0–323.0)	249.0 (225.0–268.0)	<0.001**	305.0 (282.0–334.0)	268.0 (249.0–290.0)	<0.001**
Moderate/severe	51 (100%)	16 (31.3%)	<0.001**	47 (92.2%)	27 (52.9%)	<0.001**
Degree of F0-1 of hepatic fibrosis	41 (80.4%)	47 (92.2%)	0.084	39 (76.5%)	48 (94.1%)	0.012*
Other						
UA, μmol/L	407.0 ± 95.5	322.0 ± 82.4	<0.001**	419.8 ± 98.7	350.0 ± 76.9	<0.001**
BUN, mmol/L	4.2 ± 1.0	4.1 ± 1.0	0.359	4.5 ± 1.0	4.7 ± 1.4	0.19
hs-CRP, mg/L	1.5 (0.7–3.9)	1.1 (0.5–3.4)	0.066	1.6 (0.8–4.7)	1.4 (0.6–3.8)	0.554

*P < 0.05 (intra-group difference); **P < 0.01(intra-group difference). CRD, calorie-restricted balanced diet; KD, ketogenic diet; BMI, body mass index; WC, waist circumference; HC, hip circumference; WHR, waist to hip ratio; WHtR, waist-to-height ratio; TC, total cholesterol; TG, triglycerides; LDL-C, low--density cholesterol; HDL-C, high-density cholesterol; ALT, alanine aminotransferase; AST, aspartate aminotransferase; GGT, gamma-glutamyl transferase; FBS, fasting blood sugar; FINS, fasting insulin; HOMA-IR, insulin resistance index; LSM, liver stiffness measurements; CAP, controlled attenuation parameter; UA, uric acid; BUN, blood urea nitrogen; hs-CRP, high-sensitivity C-reactive protein.

### 3.3 Effects on anthropometry and body composition

**Figure 3:**
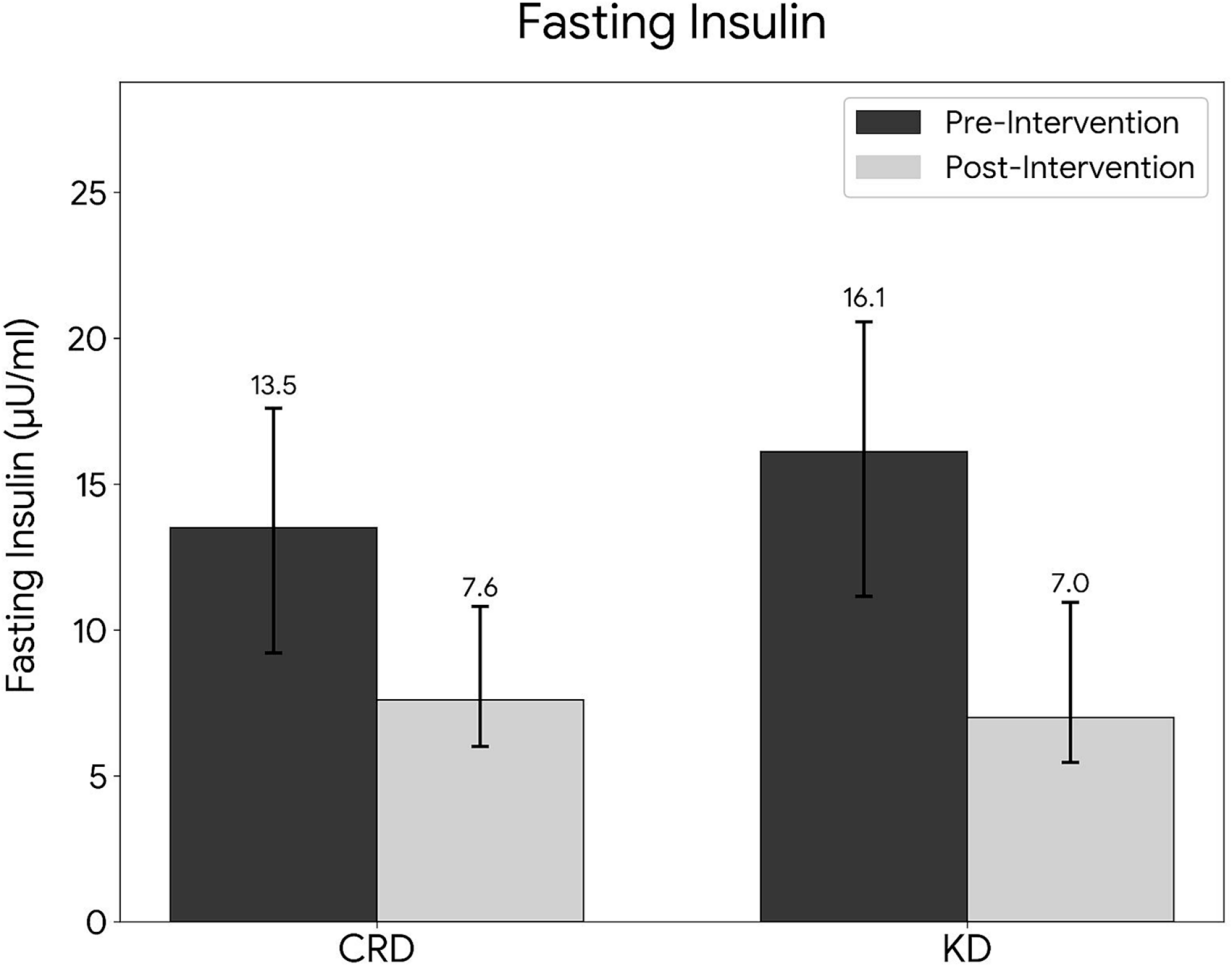
Comparison of muscle mass reduction between KD and CRD groups.

Relative to the CRD group, the KD group demonstrated significantly greater changes in BMI (*p* < 0.001, Cohen’s d = 1.00), HC (*p* < 0.01, Cohen’s d = 0.59), percent body fat (*p* < 0.001, Cohen’s d = 0.28), and visceral fat grade (*p* < 0.001, Cohen’s d = 1.07). The reduction in muscle mass was significantly greater in the KD group than in the CRD group (median Δmuscle mass: 3.0 kg (IQR: 2.2–3.9) vs. 1.8 kg (IQR: 0.9–3.1); *p* < 0.001, Cohen’s d = 0.59, Figure 3). The KD group also showed greater improvements in WC (*p* < 0.05, Cohen’s d = 0.44), WHtR (*p* < 0.05, Cohen’s d = 0.44), and fat mass (*p* < 0.05, Cohen’s d = 0.56). Changes in body weight and WHR did not differ significantly between groups (both *p* > 0.05, Cohen’s d = 0.05 and 0.02, respectively) (Table 2).

To further explore the differential effect on muscle mass, we performed subgroup analyses stratified by sex and baseline BMI (Table 4). The greater muscle loss associated with KD versus CRD was consistently observed across all subgroups, with no significant interactions detected (sex: *P* for interaction = 0.52; BMI: *P* for interaction = 0.35). In the BMI ≥ 30 kg/m^2^ subgroup, the magnitude of muscle loss was numerically larger (KD: −3.3 ± 1.5 kg vs. CRD: −2.1 ± 1.2 kg; mean difference −1.2 kg, 95% CI: −1.8 to −0.6), suggesting that individuals with higher baseline obesity may warrant closer attention for muscle preservation during KD implementation.

**Table 4:** Subgroup analysis of muscle mass change (Δ, kg).

Subgroup	*n* (CRD/KD)	ΔCRD	ΔKD	*p*-value	*P* for interaction
Gender					0.234
Male	29/27	−2.7 ± 2.3	−4.8 ± 3.7	0.006**	
Female	22/24	−1.6 ± 0.9	−2.6 ± 0.9	0.001**	
BMI, kg/m2					0.814
<30	16/27	−1.9 ± 1.6	−3.0 ± 1.1	0.003**	
≥30	35/24	−2.6 ± 2.2	−3.9 ± 3.3	0.052	

Δ values represent change from baseline to week 12 (week 12 – baseline); p values are for between-group comparisons within each subgroup (KD vs. CRD). P for interaction was derived from a general linear model including group, subgroup, and their interaction term, indicating whether the treatment effect differs across subgroups. *p < 0.05; **p < 0.01. CRD, calorie-restricted balanced diet; KD, ketogenic diet; BMI, body mass index.

### 3.4 Effects on metabolic and laboratory parameters

**Figure 4:**
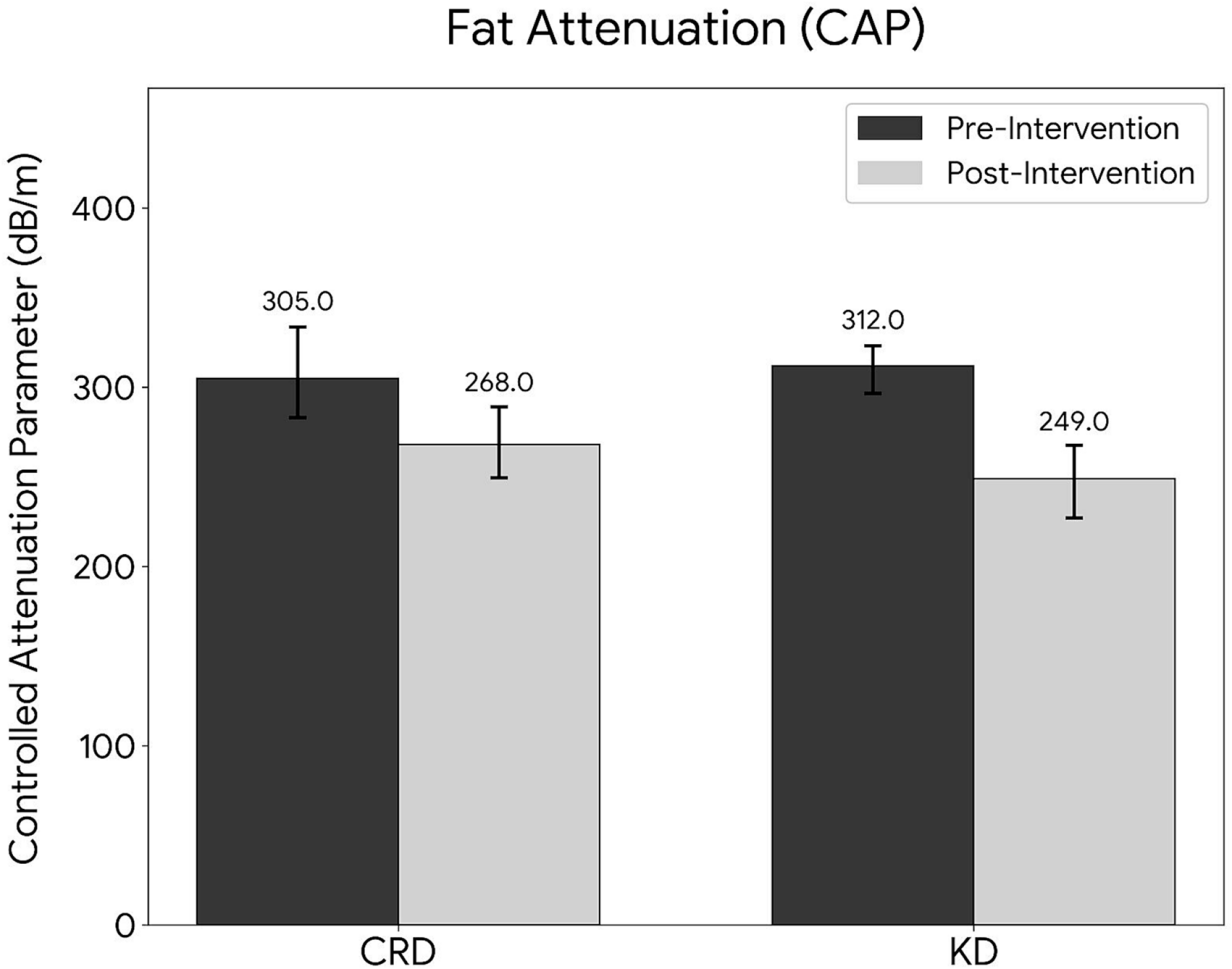
Comparison of fasting insulin reduction between KD and CRD groups.

The KD group experienced a significantly larger decrease in FINS levels compared with the CRD group (median ΔFINS: 6.4 μIU/mL (IQR: 3.4–13.1) vs. 4.7 μIU/mL (IQR: 1.4–9.2); *p* < 0.05, Cohen’s d = 0.37, Figure 4). However, after adjusting for baseline fasting insulin by ANCOVA, the between-group difference was attenuated and no longer reached statistical significance (*p* = 0.218). This inconsistency may reflect the modest effect size (Cohen’s d = 0.37) and the influence of baseline variability; the direction of the effect remained consistent with greater reduction in the KD group. Detailed ANCOVA results are provided in Supplementary Appendix 2. No significant between-group differences were observed for ALT, AST, GGT, TC, TG, LDL-C, HDL-C, FBS, UA, BUN, or hs-CRP (all *p* > 0.05, Cohen’s d ranging from 0.05 to 0.26) (Table 3). HOMA-IR showed a trend toward greater reduction in the KD group (median *Δ*: 1.6 (IQR: 0.8–3.6) vs. 1.2 (IQR: 0.3–2.7); *p* = 0.071, Cohen’s d = 0.34), suggesting a potential mild clinical effect despite not reaching statistical significance.

The comparison of pre- and post-treatment outcomes was presented in Table 3. At week 12, both groups showed significant reductions from baseline in CAP, LSM, WC, HC, WHtR, WHR, body fat percentage, fat mass, muscle mass, visceral fat grade, ALT, AST, GGT, TC, TG, FBS, UA, LDL-C, FINS, HOMA-IR, and the number of moderate-to-severe fatty liver cases (*p* < 0.05) (Table 3).

**Figure 5:**
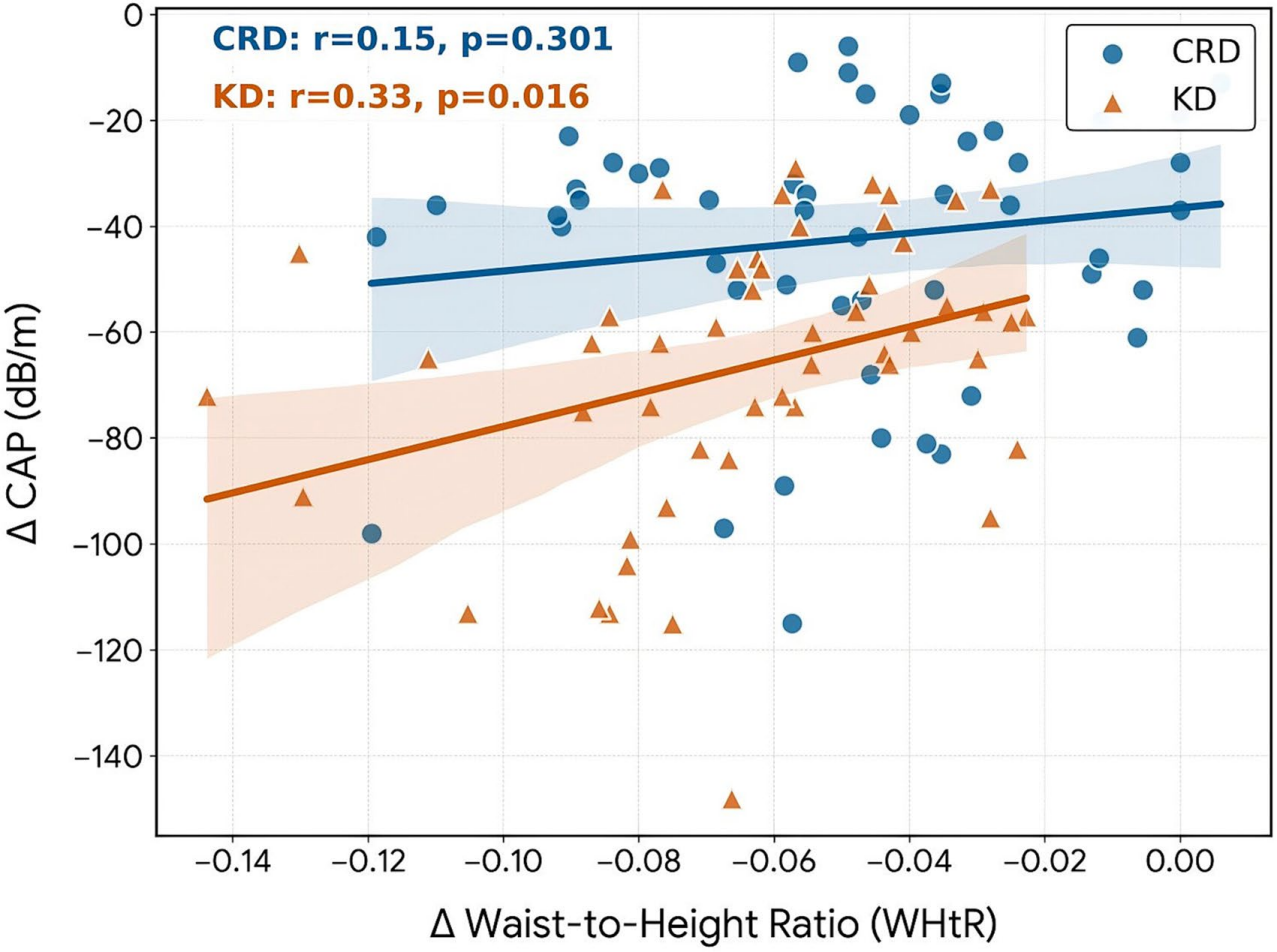
Correlation between changes in waist-to-height ratio (*Δ*WHtR) and changes in hepatic steatosis (ΔCAP).

To examine the relationship between changes in central obesity and hepatic steatosis, we performed Spearman correlation analysis between ΔWHtR and ΔCAP. This correlation was stronger in the KD group (r_s_ = 0.33, *p* = 0.016) than in the CRD group (r_s_ = 0.15, *p* = 0.301), suggesting that the reduction in central adiposity was more closely linked to hepatic fat improvement in patients following the ketogenic diet (Figure 5).

### 3.5 Adherence to dietary interventions

Adherence was assessed using a structured 0–10 point scoring system, calculated weekly by clinical dietitians based on 24-h dietary recalls, food models, and urinary ketone levels (for the KD group). The median adherence score was 8.29 (IQR: 7.71–9.14) in the KD group and 8.86 (IQR: 7.71–9.14) in the CRD group, with no statistically significant difference between groups (*p* = 0.24). These findings indicate that adherence was high and comparable across both dietary interventions, suggesting that differential compliance is unlikely to have biased the study results.

### 3.6 Adverse events

Four participants reported hunger during the initial ketogenic phase, two reported ketosis rash and one developed “keto flu.” No deaths or serious adverse events were recorded during the trial.

## 4 Discussion

In a 12-week study, we found that the KD was more effective than the traditional calorie-restricted diet in reducing hepatic steatosis. Moreover, the KD group showed a greater reduction in the number of patients with moderate to severe fatty liver disease after intervention. Our findings align with three recent studies, demonstrating that both ketogenic and carbohydrate-restricted diets effectively reduce hepatic fat in patients with fatty liver disease, with benefits extending to metabolic health (24–26). Other studies indicate that even short-term isocaloric low-carbohydrate diets (4 days) can rapidly reduce hepatic fat by 35% in overweight men (27), with this effect being independent of weight changes. More importantly, the ketogenic diet significantly improved insulin sensitivity, as evidenced by a marked decrease in fasting insulin levels. Additional research corroborates these observations (28–30), demonstrating that carbohydrate restriction helps improve hyperinsulinemia. These metabolic benefits are unrelated to weight loss.

We have established that the ketogenic diet can significantly reduce hepatic steatosis. This finding holds clinical importance, as liver fat accumulation is a defining characteristic of metabolic dysfunction-associated fatty liver disease (MASLD) and serves as a primary contributor to the disease’s progression. The results of this study align with prior research; the Cunha team previously demonstrated that a two-month very low-carbohydrate diet outperformed a conventional low-carbohydrate diet in decreasing proton density fat fraction (PDFF) and visceral fat area (14). Significant intra-group LSM improvement was noted in both groups at 12 weeks; however, there was no statistically significant difference between the groups. This finding contrasts with some reported dietary interventions aiming to enhance fibrosis, possibly due to temporal dynamic variations in steatosis and fibrosis resolution (24, 31, 32). Hepatic steatosis is a swift and reversible metabolic anomaly, whereas fibrosis entails gradual extracellular matrix restructuring that may persist for months or even years post elimination of the injurious trigger. Moreover, one must consider the technical constraints of LSM, as substantial fat accumulation alone can result in elevated LSM. The comparable LSM values between the two groups may result from a combination of reduced liver stiffness due to fat regression counteracting the stiffness maintained by ongoing fibrotic changes. The notable improvement in LSM within this cohort implies a beneficial effect of both dietary protocols on liver stiffness. The absence of intergroup distinctions does not signify treatment ineffectiveness; instead, it indicates that reversing fibrosis necessitates sustained metabolic enhancements lasting beyond 12 weeks. Subsequent investigations should incorporate extended follow-up periods of ≥12 months, alongside gold standard techniques like histological biopsies or MRI-PDFF, to ascertain whether the fat reduction prompted by the ketogenic diet (KD) can ultimately lead to fibrosis regression.

Body composition: The ketogenic diet significantly improved central obesity measures like waist circumference, WHtR and visceral fat grade. Of particular note is the striking correlation between WHtR and hepatic fat reduction. Previous studies have shown that WHtR serves as a superior predictor of metabolic syndrome and hepatic steatosis (33, 34). The observed significant positive correlation between ΔWHtR and ΔCAP in this study further expands previous cross-sectional findings, demonstrating that changes in WHtR during dietary intervention are parallel to improvements in hepatic steatosis. This dynamic relationship highlights the clinical value of WHtR as a simple, non-invasive biomarker for monitoring treatment responses in patients with MASLD (35). Notably, the correlation was stronger in the KD group than in the CRD group, suggesting that the rapid metabolic effects of carbohydrate restriction may enhance the coupling between visceral fat reduction and hepatic fat clearance. Multiple large-scale studies have confirmed WHtR’s excellent predictive performance for MASLD across different populations (36–39), further supporting its reliability as a clinical marker. This is consistent with our findings, showing a positive correlation between the decrease in WHtR and the improvement in MASLD. However, in the KD group, muscle mass decreased more significantly than in the CRD group. A randomized controlled trial (RCT) demonstrated that the ketogenic diet resulted in a marked reduction in muscle mass during the fourth week of intervention in healthy adults (40). Another study involving overweight women with polycystic ovary syndrome (PCOS) revealed muscle loss following the ketogenic intervention (41). Since muscle is critical for metabolic health and glycemic control, muscle loss may partially offset long-term benefits of improved insulin sensitivity (42). A meta-analysis involving 33 studies found that ketogenic diets per se do not necessarily cause muscle loss (43). The lack of exercise regimen in this study may be the reason for this effect, and resistance training helps to increase muscle mass. The comparable adherence across groups further supports the robustness of our findings, as varying levels of adherence are unlikely to explain the observed differences in hepatic steatosis and metabolic parameters.

Not all metabolic parameters improved after the intervention. We found no significant differences in the changes of blood glucose parameters (FBS, HOMA-IR), liver enzymes and lipid parameters among the groups. Notably, while the primary analysis demonstrated a significant reduction in fasting insulin levels in the ketogenic diet group, this effect was attenuated and no longer statistically significant after adjustment for baseline values through analysis of covariance. This discrepancy may reflect the smaller effect size and the influence of baseline variability, suggesting that although the insulin-lowering effect of the ketogenic diet is consistent in direction, it is less pronounced compared to its improvements in hepatic steatosis and body composition. Larger-scale studies are required to confirm this potential benefit. This may be due to short intervention time, near-normal baseline values, or organ specific effects of KD (targeting hepatic steatosis and insulin secretion rather than systemic metabolism). A meta-analysis showed that ketogenic diets can improve triglycerides, blood pressure, body weight and glycemic control, but may increase total cholesterol and LDL-C, which should be considered (44). However, we did not see such increase due to diet composition or variation among participants. Other than carbohydrate restriction, diet quality and composition (fat and protein sources, relative intake of saturated, monounsaturated and polyunsaturated fat) may affect metabolic response, which also causes heterogeneity in studies (45).

The ketogenic diet’s specific improvement of hepatic steatosis and fasting insulin likely arises from multiple metabolic adaptations. The reduction in fasting insulin reflects alleviation of hyperinsulinemia, which is a principal driver of hepatic *de novo* lipogenesis. By severely restricting carbohydrates, a ketogenic diet decreases substrate availability and circulating insulin levels, thereby suppressing hepatic de novo lipogenesis. This process downregulates lipogenic transcription factors such as SREBP-1c and ChREBP, leading to reduced activity of enzymes including ACC and FASN. Concurrently, the KD enhances hepatic fatty acid oxidation and ketogenesis. These changes, together with the anti-inflammatory effects mediated by ketone bodies (e.g., *β*-hydroxybutyrate), collectively promote a rapid decline in hepatic triglyceride content, highlighting the diet’s potential in treating MASLD (28, 46). From a clinical perspective, ketogenic diets can reduce hepatic fat and improve insulin sensitivity even without significant weight loss, supporting their use as a targeted nutritional strategy for patients with advanced hepatic steatosis and insulin resistance. However, the associated risk of muscle loss must be addressed through multimodal interventions, including resistance training and adequate protein intake, to maximize metabolic benefits while preserving muscle mass. This approach is supported by recent consensus statements on nutritional management of MASLD.

This study has several limitations that should be considered when interpreting the findings. First, the non-randomized, patient selection design may introduce selection bias, as individuals with greater motivation or healthier lifestyle habits might prioritize the ketogenic diet. Although baseline characteristics were comparable across groups and adherence showed no significant differences, unmeasured confounding factors (e.g., physical activity levels, dietary quality beyond macronutrient composition, and baseline metabolic severity) could not be fully controlled. Second, the study had a relatively small sample size, short intervention duration (12 weeks), and lack of long-term follow-up data, which limited generalizability and precluded conclusions regarding the durability of observed effects. Third, the use of bioelectrical impedance analysis (BIA) for body composition assessment inherently has limitations in accuracy compared to reference standards. Future research would benefit from prospective, randomized controlled trials with larger sample sizes, extended intervention periods, and long-term follow-ups. Such studies should employ more precise body composition measurements, such as dual-energy X-ray absorptiometry (DXA) or magnetic resonance imaging (MRI), and explore strategies like protein supplementation and resistance training to mitigate muscle loss, thereby maximizing the metabolic benefits of ketogenic diets while preserving lean body mass.

## 5 Conclusion

In summary, this study demonstrates that a ketogenic diet constitutes an effective interventional strategy for patients with metabolic dysfunction-associated fatty liver disease (MASLD): even in the absence of substantial weight loss, it can markedly reduce hepatic fat accumulation and improve insulin sensitivity. However, the attendant risk of skeletal muscle loss warrants caution in clinical application. Combining a ketogenic dietary approach with targeted muscle-preserving strategies—such as resistance training and adequate protein intake—should be considered. These findings provide a foundation for personalized nutritional therapy for MASLD, underscore the differential effects of distinct dietary patterns, and support the implementation of more precise, individualized treatment regimens in clinical practice.

## Data Availability

The raw data supporting the conclusions of this article will be made available by the authors, without undue reservation.
