# Supplementary material for: The effects of ketogenic diet and calorie-restricted diet on metabolic dysfunction-associated steatotic liver disease: a retrospective study
Source: Front Nutr. 2026 May 5;13:1790674. doi: 10.3389/fnut.2026.1790674 (PMC13183548; doi:10.3389/fnut.2026.1790674)
Supplement: Supplementary file 1 [file Table_1.docx]

****Dietary Adherence Recording and Scoring Form****

**Patient ID:** ________

| **Assessment Item & Criteria** | **Mon** | **Tue** | **Wed** | **Thu** | **Fri** | **Sat** | **Sun** |
| --- | --- | --- | --- | --- | --- | --- | --- |
| **1. Core Macronutrient Adherence** |  |  |  |  |  |  |  |
| **2. Food Choice Quality** |  |  |  |  |  |  |  |
| **3. Meal Regularity** |  |  |  |  |  |  |  |
| **4. Self-Monitoring Behavior** |  |  |  |  |  |  |  |
| **5. Overall Compliance** |  |  |  |  |  |  |  |
| **Daily Score Subtotal** |  |  |  |  |  |  |  |
| **Weekly Mean Score** | **(Total/70) × 10 =** | | | | | | |
| **Dietitian Review** |  | | | | | | |

**Scoring Instructions (for Dietitian Use):**

**Daily Scoring: Each item is scored as "fully achieved = 2 points", "partially achieved = 1 point", and "not achieved = 0 points". Maximum daily score = 10 points.**

**Adherence Criteria: A weekly mean score ≥7 is defined as good adherence.**
