# Supplementary material for: The effects of ketogenic diet and calorie-restricted diet on metabolic dysfunction-associated steatotic liver disease: a retrospective study
Source: Front Nutr. 2026 May 5;13:1790674. doi: 10.3389/fnut.2026.1790674 (PMC13183548; doi:10.3389/fnut.2026.1790674)
Supplement: Supplementary file 2 [file Table_2.docx]

Supplementary Appendix 2**. ANCOVA results for key secondary outcomes adjusting for baseline values.**

| **Indicator** | **P_value_ANCOVA** | **F_value_ANCOVA** | **Adj_Mean_Delta_Group_A** | **SE_Group_A** | **Adj_Mean_Delta_Group_B** | **SE_Group_B** |
| --- | --- | --- | --- | --- | --- | --- |
| **CAP(dB/m)** | 0.000** | 20.977 | -43.681 | 3.293 | -65.084 | 3.293 |
| **Muscle mass,kg** | 0.002** | 9.642 | -2.235 | 0.302 | -3.563 | 0.302 |
| **FINS(µU/ml)** | 0.218 | 1.536 | -6.722 | 0.634 | -7.838 | 0.634 |
